# Towards a Holistic Framework for the Evaluation of Emergency Plans in Indoor Environments

**DOI:** 10.3390/s140304513

**Published:** 2014-03-06

**Authors:** Emilio Serrano, Geovanny Poveda, Mercedes Garijo

**Affiliations:** Department of Telematic Systems Engineering, Polytechnic University of Madrid, Madrid 28040, Spain; E-Mails: gpoveda@gsi.dit.upm.es (G.P.); mga@gsi.dit.upm.es (M.G.)

**Keywords:** emergency plans, ambient intelligence, ambient intelligence simulation, agent-based social simulation, game engines, sensors simulation

## Abstract

One of the most promising fields for ambient intelligence is the implementation of intelligent emergency plans. Because the use of drills and living labs cannot reproduce social behaviors, such as panic attacks, that strongly affect these plans, the use of agent-based social simulation provides an approach to evaluate these plans more thoroughly. (1) The hypothesis presented in this paper is that there has been little interest in describing the key modules that these simulators must include, such as formally represented knowledge and a realistic simulated sensor model, and especially in providing researchers with tools to reuse, extend and interconnect modules from different works. This lack of interest hinders researchers from achieving a holistic framework for evaluating emergency plans and forces them to reconsider and to implement the same components from scratch over and over. In addition to supporting this hypothesis by considering over 150 simulators, this paper: (2) defines the main modules identified and proposes the use of semantic web technologies as a cornerstone for the aforementioned holistic framework; (3) provides a basic methodology to achieve the framework; (4) identifies the main challenges; and (5) presents an open and free software tool to hint at the potential of such a holistic view of emergency plan evaluation in indoor environments.

## 1. Introduction

An emergency plan is an exhaustive information guide that contains the actions and procedures that people will follow when an emergency occurs. Its main goal is to generate, in a systematic manner, a set of instructions that allow interested agents to reduce and control the effects produced by emergency events, such as fires, earthquakes and terrorist attacks. Emergency action plans can help people to remain safe in many situations. Traditionally, these plans have been validated using drills. In addition to their cost, drills present a number of shortcomings, such as not covering crowd conditions in which people may experience human behaviors, such as panic. Several recent catastrophes, including those in the Madrid Arena [1] and the Brazilian Santa Maria [2], have demonstrated the necessity of improving emergency plan strategies.

Ambient intelligence (AmI) is an emerging discipline in information technology in which people are empowered through a digital environment that primarily consists of sensors and devices connected through a network [3]. AmI is based on inherent aspects, such as artificial intelligence, human-computer interaction, sensors and ubiquitous computing. The main goal of AmI is to offer a digital environment in which it is possible to support the cooperation of devices, services and people. AmI allows creating sensitive, adaptive and responsive scenarios that interact with users seamlessly by providing them with implicit and unobtrusive interaction paradigms.

One of the most promising fields for AmI is the implementation of intelligent emergency plans in which it is possible to advise people in an intelligent manner when an emergency situation takes place in dynamic and complex scenarios. Putting decisions into action and resolving situations caused by emergency events require the integration of a set of techniques and elements that enable intelligent evacuations to be conducted. Inasmuch as AmI is meant to predict users' needs and react accordingly, this is a very suitable approach to support all the features required in an intelligent emergency plan and to allow for the collaboration and coordination strategy to improve response efforts in emergency situations. Similar works have addressed providing appropriate, updated and accessible information to all people in emergency situations [4], developing intelligent emergency algorithms to calculate the emergency plan dynamically [5,6] and improving sensors or their management to detect the symptoms of emergencies [7,8].

AmI systems are traditionally assessed by using living labs, which refers to an approach that represents a user-centric methodology for sensing, prototyping, validating and refining complex solutions in evolving real-life contexts [9]. Living labs use an infrastructure that enables developers to emulate final physical spaces, such as houses or buildings, with multiple ubiquitous devices connected and distributed across a network. Although the use of living labs is always desirable, they have the same problems as drills. In AmI research, there has been a trend of using simulations in addition to the hegemonic assessment method, living labs. However, common shortcomings include the following: (1) these simulations are usually “closed” and for a specific service, *i.e.*, they cannot be parameterized to adapt them to other cases beyond the studied case; (2) the experiments are not reproducible, *i.e.*, the information given about how the authors have built the simulations is insufficient to repeat them; and, most importantly; (3) the source code is rarely given.

Over the last few years, computer-based simulations (CBS) have become a useful part of modeling many natural and human systems [10]. CBS have enabled emergency committees to improve the performance and efficiency of many emergency plans while decreasing the limitations of regular drills. By using CBS, it is possible to: (1) reduce the cost of experimentation; (2) control the variables and parameters of the experiment; and (3) control the time of the experiment. Several research works based on CBS have been proposed and developed to validate different types of emergency plans. Among those, the use of agent-based social simulation (ABSS) is one of the most representative research streams.

ABSS is a computer-assisted simulation technique used to model artificial societies populated with multiple autonomous entities, called agents, which act autonomously by employing some knowledge or representation of their beliefs, desires and intentions. ABSS is an innovative approach to open questions in a wide range of scientific domains, including economics, biology, chemistry, ecology and sociology. One of the main advantage of using ABSS to evaluate emergency plans is that it allow components of a final agent-based architecture to be connected in the simulation to be tested [11].

In contrast with the large number of research projects and ABSS platforms in which emergency evacuation is a recurrent topic [12] and the also vast number of emergency planning approaches that are studied by simulations [13]: (1) the hypothesis this paper defends is that little interest has been shown in formally describing the key elements that these simulators must include in this domain. Most importantly, contributions that focus on some of these elements are usually presented without making the implementations available for researchers. This hinders researchers from interconnecting these contributions with other tools to achieve a holistic framework for emergency plan assessment. To support this hypothesis, over 150 simulators have been considered in different relevant scopes: general ABSS frameworks, game engines, simulators for the emergency domain and AmI simulators.

In addition to discussing the hypothesis explained above: (2) this paper also defines the authors' idea of the main modules required in such a holistic framework. Semantic web technologies, which concentrate on declarative forms of knowledge representation, are considered here as the cornerstone of the holistic framework. The reason is that the use of these technologies would allow (or at least facilitate) different implementations to understand each other. Even when this is not possible, researchers would always have a knowledge repository to use in their new implementations.

Furthermore, the paper: (3) proposes a methodology to develop and use a holistic framework based on the development of AmI systems; (4) discusses the main challenges; and (5) presents an open-source tool to achieve a simplistic version of the proposed holistic framework. The authors expect this paper to be a research proposal about the most efficient way of completing these modules by reusing existing works if possible and how to facilitate its future use and extension.

The paper outline is as follows. After presenting the background that supports the hypothesis stated in this paper in Section 2, the main components identified for the holistic framework are given in Section 3. A methodology for the development of this holistic framework and the main challenges are described in Section 4. A preliminary implementation is described in Section 5. Some illustrative experimental results are discussed in Sections 6 and 7 concludes.

## 2. Background

This section describes a number of possible frameworks to evaluate emergency plans using simulations in four main categories: ABSS frameworks, game engines, emergency domain simulators and AmI simulators.

### 2.1. Agent-Based Social Simulation Frameworks

An interesting wiki [14] on platform comparisons presents the large number of frameworks available for general ABSS. This wiki currently lists 81 frameworks with information that is important for the purpose of this paper, such as the license. To the best of the authors' knowledge, none of these simulators gives abstract mechanisms for simulating emergency plans. In general, the most popular ABSS frameworks, such as NetLogo [15], MASON[16] and Repast [17], do not offer tools to build a realistic environment model with the capacity to perform emergency-plan evaluations. Nevertheless, these platforms can supply interesting support for developing more abstract resources. In this vein, Dawson *et al.* [18] use NetLogo to study emergency plans for flood incidents.

### 2.2. Game Engines

The use of general game engines is also very common when there is a need for modeling a real environment with agents moving through it. This is an essential requirement for emergency simulators, which is usually solved in these platforms, every time better and more automatically, by using navigation meshes and pathfinding methods. Barbosa *et al.* [19] propose the use of virtual simulation to evaluate emergency operating procedures in the nuclear industry through reusing a game engine platform. In this work, the real environment may be virtually modeled, and people are able to virtually navigate and interact with each other. Szymanezyk *et al.* [20] adapt popular video-game technology for an agent-based crowd simulation framework in an airport terminal. There is a wiki [21] that details the main properties of a large number of these engines. Strangely enough, this wiki also lists exactly 81 game engines, the same number of ABSS frameworks mentioned above. Some of the most popular engines are Unity [22] (proprietary and written in C++), GamePlay3D [23] (under the Apache license and written in C++) and JMonkey [24] (under the BSDlicense and written in Java). Although these engines are incredibly useful for our goal, they lack important elements, such as a model of simulated sensors or abstract behaviors for the users modeled. Some works overcome these limitation by considering real users as players to study their response in a simulated emergency [25]. Moreover, the parameterization of simulations built by these engines is still complex; thus, they are only employed for closed simulations, *i.e.*, those in which changing the scenario or the elements requires rebuilding the engine.

### 2.3. Emergency Domain Simulators

A number of domain-specific simulators address the emergency domain in indoor environments [26]. Some examples are EVACNET4 [27], WAYOUT [28], STEPS [29] and SimWalk [30]. These works give excellent tools to model emergencies, but: (1) they do not contemplate the inclusion of AmI features to assess an intelligent plan, such as a sensor model and actuators to guide users intelligently; (2) they tend to ignore the multi-agent paradigm, which, as stated in the Introduction, is an intuitive manner of studying complex adaptive systems; and, more importantly; (3) the code is often not available, and thus, researchers cannot extend, reuse or simply learn from it.

### 2.4. Ambient Intelligence Simulators

Campillo-Sanchez *et al.* [31] review six different simulators devoted to evaluating general AmI systems, including UbiWise [33], TATUS [34] and UbiReal [35]. Although they enable developers to design realistic environments, the user role has to be played by a real user interacting with the simulator. Therefore, it is not possible to conduct batches of experiments to obtain statistically significant results. Moreover, the authors do not give the source code, thereby hindering researchers from reusing modules of these works. This is also the case of the simulators proposed by Tang *et al.* [36] and Diewald *et al.* [37], which, although they are significant contributions for the AmI field, do not provide researchers with open implementations. Hence, to the best of the authors' knowledge, there is no open simulator that focuses on AmI general evaluation besides the one extended, released and presented in this paper.

### 2.5. Partial Conclusion

These works demonstrate that there are numerous attempts to assess emergency plans by simulations, and excellent contributions have been proposed. However, there is no “most complete” tool, and most of the time, it is not possible to reuse and combine components of different frameworks. Further evidence of this can be found by consulting the EU Research Projects on the CORDIS (Community Research and Development Information Service) website [38], where a search with the keywords “emergency” and “simulation” returns over 8,000 results of funded projects. As a result, without a true interest in sharing and interconnecting works, researchers in this domain are constantly damned to reinvent the wheel.

## 3. Main Components of the Holistic Framework

**Figure 1.**
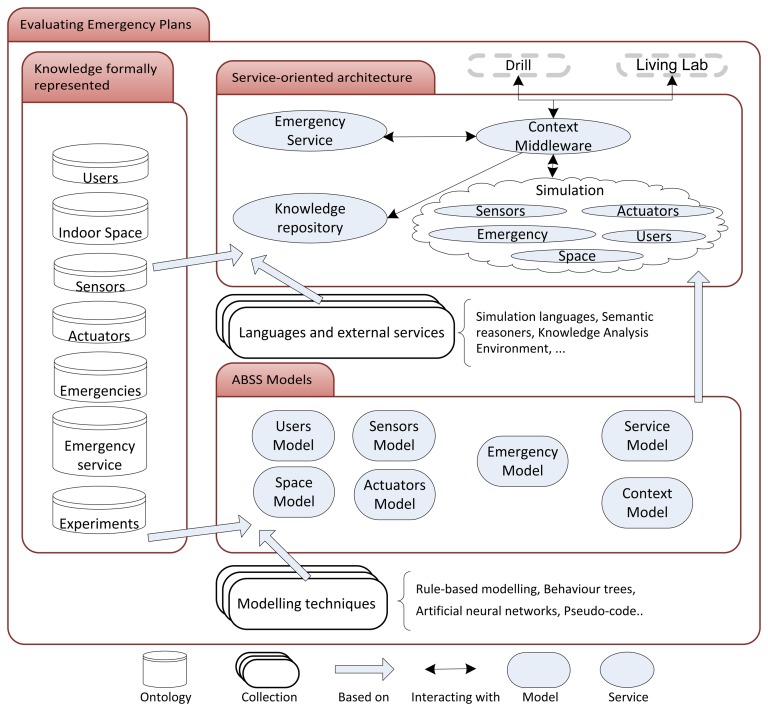
A holistic framework for the evaluation of emergency plans.

This section describes the proposed holistic framework for the evaluation of emergency plans. In an attempt to narrow down this challenging problem, the framework only addresses indoor environments. Figure 1 depicts our vision of the holistic framework. The formally represented knowledge, on the left side of the figure, is the cornerstone of the framework. This knowledge is the basis for the other two main components: the ABSS models and a service-oriented architecture. These three components are explained and justified below.

### 3.1. Formally Represented Knowledge

As stated above, the basis of the framework is formally represented knowledge and, more specifically, the use of ontologies. There are several reasons to develop ontologies [39]. They allow a common understanding of the structure of information to be shared among people or software agents. For this scope, this means that if tools are based on the use of ontologies, these are more likely to be understood by developers and to be connected successfully with other tools (whether they are developed by other researchers or not). Furthermore, ontologies enable the reuse of domain knowledge and, as stated in Section 2, given the number of projects and software packages that address this problem, reusing components is a must. Moreover, ontologies make domain assumptions explicit and enable experts in emergency plans (who may not have expertise in computer science) to verify these assumptions and change them if needed. Ontologies also separate the domain knowledge, which is very similar in the vast literature of emergency-plan evaluation, from the operational knowledge, which is considerably different in these works. Last, but not least, there are a number of powerful mechanisms developed over ontologies that can be used for the remaining components of the framework (such as the SPARQL [40] for queries or SPINfor rules and constraints [41]).

The following necessary basic ontologies have been identified:

- *Users*. The users are the humans who need to be saved in case of an emergency by the final AmI system. There are a number of works that address ontologies that model AmI users [42], which cover some of the concepts (or classes in OWL [43] terminology) necessary for the emergency evacuation domain. Some examples are the personal context (such as location, state and time) and personal information (such as contact information, disabilities and physical information). Obviously, navigation in indoor environments is highly challenging for the severely visually impaired [44]. These ontologies need to be adapted to include more knowledge relevant for the domain, such as knowledge about the routes or the adoption of special roles in emergencies, such as “leader”. Of course, this knowledge may not be available in the final system if sensors cannot perceive it or if it cannot be provided *a priori*. However, even in these cases, a user's ontology is very valuable to study the main human aspects that can affect the emergency plan and, therefore, to test the robustness of an emergency plan by considering a great variety of user populations.
- *Indoor space*. Users, as other elements of the AmI system, are immersed in an environment (an indoor space for the considered domain). An AmI system is basically a system that uses processing units, sensors and actuators distributed in the environment to support humans in their daily lives [45]. In the emergency management case, the environment limits, among others, people's speed and the routes that can be used for evacuations. Again, it is easy to find ontologies for indoor spaces [46] with relevant concepts, such as doors, stairs, elevators or fire doors, but it is more difficult to find concepts that are required for a variety of possible emergencies (drains for floods, for example) or necessary to conduct a realistic simulation (the combustibility of the furniture, for instance).
- *Sensors and actuators*. Sensors and actuators are the artifacts that are used to, respectively, capture the events from and act on the environment in which the humans are living, working, driving or learning, for example [47]. Compton *et al.* [48] revise different semantic specifications of sensors and the concepts they usually include, such as accuracy, frequency, field of vision, units of measurement, location and operating conditions. Regarding the actuators, the possible notification devices in case of emergency have been extensively discussed [49] (they include phones, the web, e-mails and SMSs).
- *Emergencies*. Another important category for which to formalize knowledge is the emergency that the service needs to address. The consideration of fires is certainly the most studied case in the emergency management of indoor environments, but there are other, less popular emergencies, such as floods or bombs. Furthermore, although it is easy to find works that simulate these emergencies, they usually lack a formalization of the knowledge that could help developers validate their correctness and extend them.
- *Emergency service*. This ontology has to cover the main concepts of the emergency service to be evaluated and that motivates the holistic framework proposed in Figure 1. Several works have tried to formalize this knowledge [50], which includes, among other concepts, scene control, medical aid, evacuation, monitoring and alerts and victim assistance. However, the interconnection among these concepts and relevant ontologies, such as the ones defined above, is not discussed.
- *Experiments*. Finally, an ontology for the experiments is required to include, at least, the simulation configuration and the main metrics contemplated for the evaluation of the service. Therefore, this ontology necessarily includes concepts of the remaining ontologies and formalizes the evaluation to be conducted by simulations, which, as shown in Section 2, are conducted for thousands of projects.

Note that all these ontologies are interconnected. Therefore, if a graph was defined with a vertex for each ontology and edges indicating that two ontologies are interconnected, the resulting graph would be complete, *i.e.*, every pair of distinct vertices would be connected by a unique edge. Some examples include the following: the location of a user is a concept of the indoor space; a fire of the emergency ontology can excite a smoke sensor; and the emergency service may indicate an escape route by a number of actuators. Therefore, although there are ambitious works in the definition of ontologies for emergency management [49], to the best of the authors' knowledge, the identification of these key ontologies is a novel contribution of the presented work. Moreover, these works hint at the difficulty of defining not only the whole holistic framework of this paper, but also this specific module.

Note also that, according to the semantic web philosophy, considering or including elements of external ontologies (concepts, roles or individuals) in the framework presented involves using URLsto link to these elements instead of replicating data. In other words, the linked data principles stated by Tim Berners-Lee [51] must be followed: (1) a URL should point to the data; (2) anyone accessing the URL should get data back; and (3) the relationships in the data should point to additional URLs with data.

### 3.2. Agent-Based Social Simulation Models

The ABSS model is a representation of the real system to be studied by simulations [52]. In this case, the real system is the AmI system for emergency management, and the model (which, in the framework proposed in Figure 1, is separated into several models) is an abstraction of this reality that has to be easily studied (or, at least, in an easier manner than treating the reality directly). As stated in the introduction, emergency management is a field in which simulations are a typical solution, because they enable researchers to study emergencies under a number of situations that cannot be reproduced in drills.

The framework demonstrates that these models are based on: (1) the ontologies of the formally represented knowledge; and (2) a modeling technique. As explained by Benjamin *et al.* [53], although ontological analysis has been demonstrated to be an effective first step in the construction of robust knowledge-based systems, the modeling and simulation community, in general, has not enjoyed the benefits of ontology-management methods and tools. Given the number of works and projects that address emergency simulations, ontologies are essential for facilitating simulation models' interoperability, composition and information exchange at the semantic level [53].

There is a clear mapping from the ontologies to the identified models. Two exceptions are the following: (1) the experiment ontology, which is not employed by the ABSS models, because it addresses the implementations of these models (see Section 3.3); and (2) the context model, which gathers, merges, interprets, reasons and stores the contextual information needed by the emergency service [54]. Although some works address isolated context ontologies [55], in this scope, in which a number of ontologies address the main aspects of the problem, most of the concepts of the context are spread through the aforementioned ontologies.

It is important to note that although there exists a clear mapping from ontologies to models, ontologies have expressive limitations that make models necessary. In the semantic web literature, these limitations are usually overcome by rule-based knowledge. Accordingly, because the holistic framework presented is based on the use of ontologies, semantic rule-based modeling is recommended as the basic modeling technique for the agents included in the different ABSS models. For example, the context model may include the following rules (written in pseudo-code inspired by SWRL [56]):

(1) $$\begin{array}{l} \begin{array}{ll} R_{1}: & \text{Sensor}(?s)\wedge \text{hasEvent}(?s,?e)\wedge \text{SmokeEvent}(?e)\wedge \\ & \text{value}(?e,1)\wedge \text{hasLocation}(?s,?l)\Rightarrow \text{DangerousLocation}(?l) \end{array}\\ \begin{array}{ll} R_{2}: & \text{Sensor}(?s)\wedge \text{hasEvent}(?s,?e)\wedge \text{MotionEvent}(?e)\wedge \\ & \text{value}(?e,1)\wedge \text{hasLocation}(?s,?l)\Rightarrow \text{LocationWithUser}(?l) \end{array}\\ \begin{array}{ll} R_{3}: & \text{User}(?u)\wedge \text{hasSmartphone}(?u,?s)\wedge \text{hasLocation}(?s,?l)\\ & \Rightarrow \text{LocationWithUser}(?l) \end{array}\\ \begin{array}{ll} R_{4}: & \text{LocationWithUser}(?l_{1})\wedge \text{closestVisibleNotificationDevice}(?n,?l_{1})\wedge \\ & \text{hasLocation}(?n,?l_{2})\Rightarrow \text{LocationWithVisibleNotificationDevice}(?l_{2}) \end{array} \end{array}$$

*R*_1_ checks for smoke events with a value of one (smoke detected) in the sensor, ?*s*, to set the location of a danger zone in ?*l*. Analogously, *R*_2_ checks for the activation of motion events to detect possible user locations. *R*_3_ considers users with smartphones, including some type of indoor location system that allows the context model to obtain the device (and user's) location. Finally, *R*_4_ obtains positions for visible notification devices (such as monitors or the aforementioned smartphones). Note that the rules contain parts that obtain relevant information for the emergency service derived from data collected through sensors, such as dangerous positions, to infer the emergency state, users' locations to consider possible crowds in the escape route and notification device positions to display escape routes calculated from those points, for example.

As ontologies, these rules have expressive limitations. For example, because of the open-world assumption, rules that attempt to enumerate individuals or properties in an ontology are not always possible [57]. Rules and inferences based on the number of individuals or property values in an ontology are not expressible in SWRL (unless OWL statements explicitly state those numbers). Therefore, with the aforementioned context model and using the same semantic rules for the emergency service, this service could not list the individuals of the *DangerousLocation* class to take them into account. On the other hand, this would be possible by using built-ins for lists, which are usable in OWL Full [43]. Another option would be to introduce the positions one at a time, as shown in the following rule that the emergency system model may consider:

(2) $$\begin{array}{ll} R_{5}: & \text{DangerousLocation}(?l)\wedge (\text{notConsideredByService})(?l)\wedge \\ & \text{EscapeRoute}(?r)\Rightarrow \text{includeForEscapeRoute}(?r,?l)\wedge \\ & \text{ConsideredByService}(?l) \end{array}$$

The example shown above illustrates that the use of semantic rules is not always appropriate or easy. Therefore, semantic rule-based modeling is desirable in the presented framework, but not necessary. Moreover, the main idea of proposing this framework is trying to integrate previous works with it, and obviously, those works may be based on other modeling techniques. The use of behavior trees [58], for instance, is very widespread for modeling agents' behaviors in video games, and they could be employed for the user models. Garcia Valverde *et al.* also propose the use of hierarchical automata to model human behaviors in AmI systems [59]. Another example is the use of cognitive BDI (belief desire intention) agents in ABSS as introduced by Caballero *et al.* [60].

This raises an important question: are the ontological components of the framework worthwhile when no semantic web technology is going to be employed at the modeling and implementation stages? Benjamin *et al.* [53] note that the design and creation of ontologies, as a previous step of ABSS modeling, allow, at least, a terminology harmonization to enable shared and clear understanding by disambiguating terminology. Furthermore, ontologies facilitate detailed analysis of objects and mapping the simulation model constraints to the specifications of real-world constraints under the specific domain. Therefore, the ideas presented in this framework are valid for a range of scenarios. More specifically, its application to emergency management in outdoor environments is straightforward by adding new elements to interconnect different indoor spaces.

### 3.3. Service-Oriented Architecture

After defining the model, in most ABSS research methodologies, there is a building stage that consists of translating the model into something that can be used by a computer (*i.e.*, programming the model) [52]. The proposed framework presented in Figure 1 demonstrates that this building stage is conducted by combining the ABSS models, the formally represented knowledge and a collection of languages and external services.

As shown in Figure 1, the resulting implementation is a service-oriented architecture (SOA), *i.e.*, a software architecture based on discrete pieces of software that give application functionality as services. Among others, design principles underlying SOA emphasize reuse, abstraction, loose coupling and composability [61]. These principles are a must for the evaluation of emergency plans, because of the number of research projects that address this issue. As in the combination of ABSS and semantic web technologies, there are works that detail the number of benefits derived from the use of these semantic technologies in SOAs [62].

Another important reason to propose an SOA is that it is one of the most widely employed paradigms for the development of AmI systems [63]. Note that unlike in most ABSS research, some of the models of the proposed framework must evolve until becoming the final AmI system. More specifically, the simulation implementation (which is composed of services for simulating sensors, actuators, users, emergencies and an environment) is employed to evaluate an emergency service and the context model that connects them. After the assessment conducted by simulations, the simulation services have to disappear, and the same context middleware has to connect the emergency system with living labs for further evaluation drills.

In addition to these three evaluation levels (simulations, living labs and drills), the framework makes possible the combination of real elements with simulated entities [12]. This approach involves the following: participatory simulations, in which scenario-guided agents and human-controlled avatars coexist in a shared virtual space and jointly perform simulations, and augmented experiments, in which an experiment is performed in real space by human subjects enhanced by the simulation [64]. Moreover, depending on the service implementation, humans can play not only the role of AmI users, but also the role of sensors. In this vein, the use of human-centric wireless sensor networks (HWSNs) is a relevant technology that has been employed to implement services focusing on reducing energy consumption [65], improving personal security [66] and urban search and rescue activities [67]. HWSNs are opportunistic networks that consist of regular sensors, information holders and human-based sensors [67] (*i.e.*, people that input information to the network and that can provide qualitative information based on quantitative and qualitative information provided by other nodes). For participatory simulations, the implementation presented in Section 5 allows avatars in simulations to be controlled by developers using a keyboard and mouse. Moreover, the avatar's viewpoint can be adopted as in a first-person game. For augmented experiments, the aforementioned implementation has been used to make mobile devices, which can be used by humans playing the role of human-based sensors or AmI users, perceive simulated entities [31] (video available on-line: [32]).

A general programming language or one of the more abstract languages to implement simulation services can be used for the construction of the architecture (Section 2 discusses plenty of alternatives). The second option is considerably more convenient, because these works already give basic elements to address this problem. For example, the generation of a realistic indoor environment and coding the possibility of making an agent go from A to B in this environment could take several person-years with a general programming language. Aside from programming languages, a number of external tools are typically required and used in the simulation of emergency plans. If the models are based on semantic rules as proposed in Section 3.2, the use of semantic reasoners, such as SPIN [41] and Jena [68], provides developers with a natural transition from the model to the implementation. Again, for the sake of reuse and extension, open and free software is recommended.

**Figure 2.**
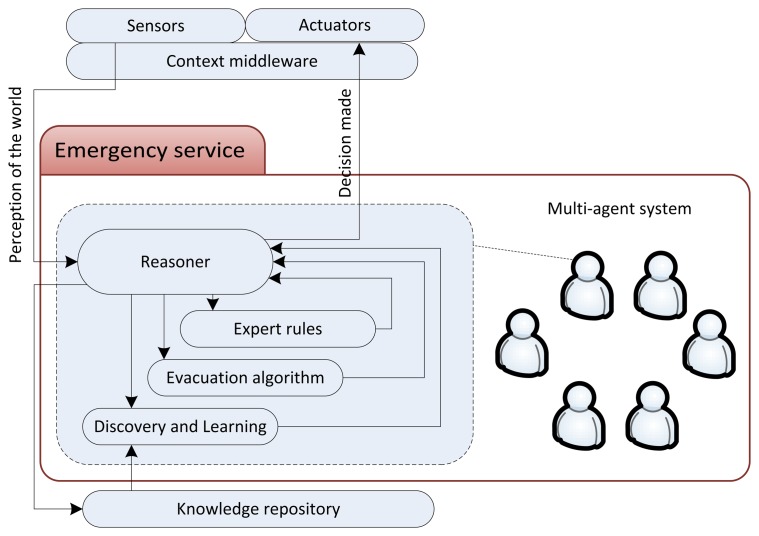
Details of the emergency service in the holistic framework for the evaluation of emergency plans.

Once more, the reader can appreciate the complexity of the research proposal this paper presents if an isolated service is considered. With this illustrative purpose, Figure 2 displays a possible emergency service. The sensors give the data perceived from the world to the context layer, and the latter has to provide the emergency service with more abstract information, such as dangerous positions and user locations (see the semantic rules proposed in Section 3.2). This service, which is usually modularized in agents in charge of zones or sub-services, needs: (1) to reason about the aforementioned information to understand that there is an emergency, its nature and its state; (2) to feed an emergency plan generator (typically an evacuation algorithm); (3) to adapt this plan with expert rules if necessary; (4) to discover and learn patterns in past experiences [69] (which come from simulation experiments) to improve the service; and (5) to communicate the decisions made to the context middleware.

## 4. Methodology and Main Challenges

**Figure 3.**
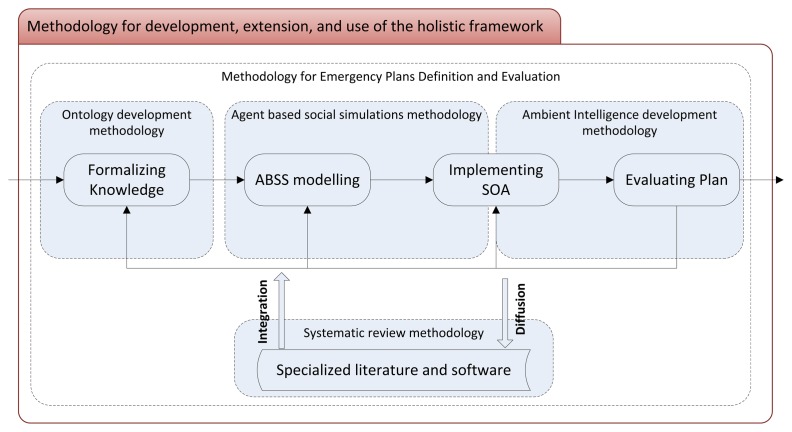
Methodology for the development, extension and use of the holistic framework. ABSS, agent-based social simulation; SOA, service-oriented architecture.

The framework explained in Section 3 describes three clear modules: the formally represented knowledge, the ABSS model and the service-oriented architecture. Because each one is based on the following, Figure 3 describes a simple flowchart with subroutines that clearly address these modules. Iterative and incremental development is necessary, as in several well-known strategies from software engineering (such as Spiral, Prototyping and any Agile software method [70]). Hence, the flowchart also describes the iterations over these three main subroutines. The fourth and, after the required iterations, final subroutine is the evaluation of an emergency plan based on the use of the holistic framework presented in this paper.

Although emergency management evaluation by means of simulations is a hot topic, as described in the Introduction, this is the first work that proposes and justifies the combination of semantic web technologies, agent-based social simulations and ambient intelligence systems. Therefore, there is not a detailed methodology for the development of such a system in the specialized literature. Figure 3 describes the main methodological research streams that have to be contemplated: (1) an ontology development methodology, such as METHONTOLOGY[71]; (2) an agent-based social simulation methodology, such as the one proposed by Gilbert and Troitzsch [52]; and (3) an ambient intelligence-development methodology, such as the formal approach proposed by Coronato and De Pietro [72]. Moreover: (4) methodologies for the emergency plans definition and evaluation are required during the whole process, because of the specific domain nature of the framework. Finally, the: (5) systematic review methodologies [73] have to be considered in an attempt to integrate available works when possible and to disseminate research results for this recurrent topic.

Although providing reference examples of methodologies for the isolated modules is easy, as shown above, the main challenge identified in the full deployment of this methodology is to include interactions among methodologies of disparate research streams. As explained above, the combination of the technologies contemplated here is an unexplored field, although some works address some of them [31,47]. Most importantly, as in any evaluation based on simulations, the ultimate question is whether the conclusions are valid for the real world or just a result of an incorrect verification and validation of the software [52]. This question is extremely challenging even when considering isolated components of the framework: For example, are the simulated users realistic? Are the sensors responding to the simulated events as they would do with real ones? If debugging traditional software is considerably complex, debugging multi-agent-based simulations and multi-agent systems in which intelligent or emergent behaviors may appear is much more complex, because a well-known feature of agent technologies is their ability to generate unpredictable, complex and emergent behaviors from very simple rules [74].

## 5. Implementation and State of the Framework

As explained above, there are a number of simulators in the literature that cover some of these modules. Unfortunately, to the best of authors' knowledge, none of them cover all the relevant modules. Particularly, no open-source simulator of pedestrian movement under both normal and emergency conditions has been found. Even considering proprietary software, the reaction to possible actuators, such as indications in panels, and the error that sensors may include are not usually considered.

Currently, UbikSim [75], a general AmI simulator, has been extended to implement different modules of the holistic framework for the evaluation of emergency plans in the indoor environments presented in this paper. Although some preliminary versions of UbikSim were distributed under an academic license [12] (until 2010), the current version has been recently documented, tested and released via GitHub (on 31 January, 2013) [75] in the scope of this research work and in collaboration with the University of Murcia (see the UbikSim license [75]). UbikSim is open and free software, and its GPLlicense [76] ensures that its extensions will remain open and free. This simulator has been employed to explain graphically possible emergencies detected after processing the data collected through artificial sensing [77], among other applications.

**Figure 4.**
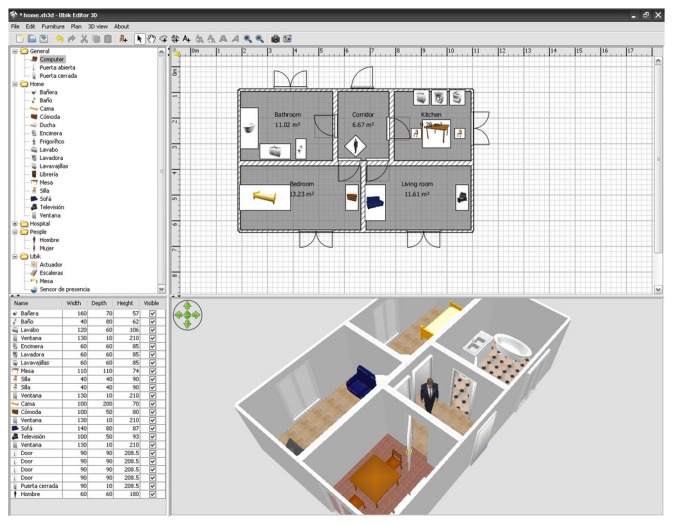
Designing an indoor space model with UbikSim.

One of the most significant improvements of this new version is that the environment model, which is a crucial factor in designing emergency plans, is realistic and parameterizable. Unlike most social simulation frameworks (see Section 2.1), which consider the space to be a toroidal grid, UbikSim allows creating realistic space models by using a drag and drop editor; see Figure 4. Unlike most simulations based on games engines (see Section 2.2), the model is parameterizable: developers cannot only modify common attributes in the elements simulated (such as the location, size, name and role), but also add any metadata needed in the simulations (such as the floor adhesiveness, furniture combustibility and maximum capacity of the stairs).

It is important to note that although the evacuation case has been a recurrent example of UbikSim's use in a number of research works (see the papers section in [75]), it lacks some important features of an emergency simulator, such as realistic models of emergencies, evacuation algorithms for the AmI system and abstract behaviors for agents in this domain, such as “lead the evacuation of this floor” or “panic”. The EscapeSim [78] library has been created to address these aspects. The border between UbikSim and EscapeSim is blurry, because when some feature is developed in the scope of EscapeSim and is considered interesting not only for the emergency domain, but also for general AmI study, this feature is integrated into UbikSim to be automatically inherited by EscapeSim.

This distinction between UbikSim and EscapeSim allows the EscapeSim library to offer remarkable usability. This simplicity of use was checked as part of the course “Agent-based social simulation” given at the Polytechnic University of Madrid. In this forum, undergraduate students were able to use EscapeSim after a few lessons to: (1) define emergency scenarios; (2) use predefined emergency management strategies; (3) implement new strategies; (4) build user profiles, such as handicapped or emergency managers; and, as described in Section 6, which was a programming assignment of this course; (5) conduct an evaluation of emergency plans though a batch of simulations.

Note that this paper is a research proposal, and we are still far from achieving the holistic framework described in Section 3. Our final purpose is to combine it (or replace it if needed) with other tools. More specifically, the definition of the ontologies required and the development of models based on semantic web technologies are still under development. On the other hand, EscapeSim in combination with UbikSim is the first AmI simulator for evaluating emergency plans that allows the interested reader to download it [78] and adapt it for her own use. Possible adaptations include creating new environments (walls and rooms may be drawn, and then, the user can drag and drop other elements, such as sensors, users or furniture) and creating new user profiles with different emergency plans. In addition to the source code of the framework [78], there is a video available on-line [79] that shows the use of EscapeSim for developing a space model and assessing different emergency plans.

## 6. Case Study

**Figure 5.**
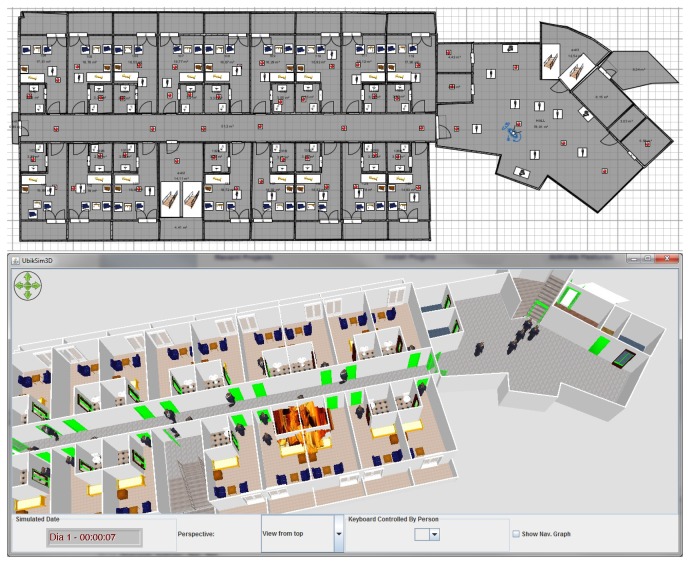
Using UbikSim for the emergency plan evaluation (the definition of the space model is in the (**top**) panel, and the caption of a simulation is in the (**bottom**) panel).

In the scope of the project *Technologies for the future of hotels* (THOFU) [80], EscapeSim has been employed to model a hotel environment and to assess emergency evacuations in this space. The top panel of Figure 5 shows the definition of the environment model with a number of points that model fire sensors that the context model uses to know the state of a fire. In the bottom panel, this figure also shows an execution of the simulation with a fire, which is started in a random position to be able to evaluate the emergency under previously unknown circumstances. By default, this simulation includes 27 users, who follow the shortest path that they receive via smartphone from the emergency service.

The simulation GUI makes it possible to study the effects of a number of what-if scenarios: What happens if the fire is near an exit? What if an exit is the closest to most of the rooms? Does the emergency plan produce crowds? One of the conclusions reached by this study was that given that the central staircases of the environment cover most rooms (see Figure 5, top), an emergency plan based on using the shortest path produces crowds, which delay the escape. Undoubtedly, these results are valid just for this specific hotel. This issue, as explained in the Introduction, is one of the reasons to study emergency plans in the scope of a specific case.

As observed above, the study of the simulation GUIis worthy. Nevertheless, the GUI tends to disappear for the evaluation in simulation research [12]. As a result, batch experiments are employed to obtain general conclusions of the emergency plan being tested. EscapeSim includes the infrastructure required for: (1) defining a set of metrics to study the plan; (2) executing a batch of simulations; (3) logging the metrics defined in (1) for each time step and each simulation; and (4) conducting statistical operations over these data to summarize the results.

Just for illustrative purposes, EscapeSim includes two basic pre-defined metrics: *PeopleInBuilding*, which is the number of users who are on the floor for each time step; and *PeopleWhoReachEmergency*, which is the number of users who are in touch with the simulated emergency at each time step. The *PeopleWhoReachEmergency* does not necessarily measure failures of vital functions in the simulated users, although the simulator allows this option if required (in addition to defining new metrics). By default, *PeopleWhoReachEmergency* is increased when a user occupies the same position as the simulated emergency and only once per user.

Additionally, several emergency plans have been implemented:

1. *Random initial wait*. This policy consists of making agents wait a random number of simulation steps (zero to 50) before starting the flight to minimize breakdowns in access to exits. As explained, by default, the service provides users with the shortest path to the closest exit.
2. *Distributing exits based on escape order*. The first user evacuated receives a path to the first exit, the second to the second and the third to the third, whereas the fourth user receives the first exit again (assuming three exits, as in the environment modeled), and so on.
3. *Avoiding the exit closest to the fire*. After calculating the Euclidean distance to the fire (specifically, to the position of the fire calculated based on the sensors excited), the closest stairway to that position is discarded for the evacuation.
4. *Avoiding routes that include positions with fire*. To improve the plan shown above, only routes that include positions with fire are ruled out. Therefore, if it is possible, the service makes use of all exits and, if more than one fire escape is risky, they are not used.
5. *Putting out the fire*. A random user is chosen as the emergency manager and led to the lobby to activate sprinklers that put out the fire. Then, the service guides this user to the closest fire escape.

**Figure 6.**
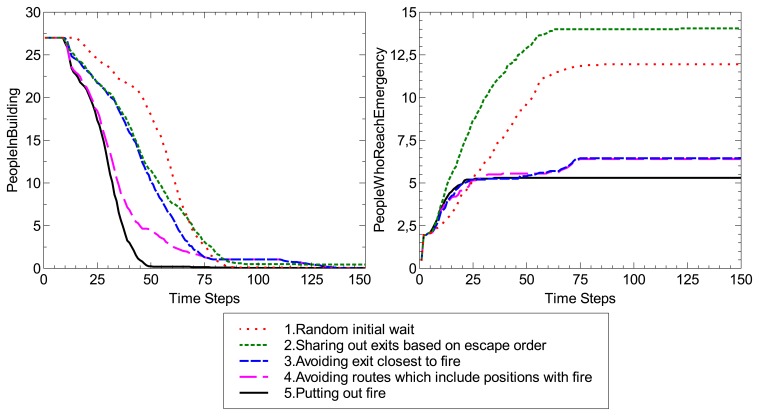
Experimental results with EscapeSim.

After executing 100 experiments with each emergency plan, the mean of the results for both metrics is displayed in Figure 6. The data are also available on-line with the standard deviation [81]. The results demonstrate that putting out the fire (fifth plan), even when it is performed by a random user who can be far from the hall, gives the best results for both metrics. The fourth plan is the next best option, which is to evacuate most of the building as fast as possible. Nonetheless, some users can remain in the building for more time than with the first and second plans, because the service tries to avoid routes in touch with the emergency. Moreover, the number of users who reach the emergency is similar to the case when the third strategy, which is much simpler, is used.

Although these experiment are just to illustrate the use of EscapeSim, these results hint at the potential of studying emergency plans by simulations and contributing with open and free software. Moreover, as explained in Section 5, the usability of EscapeSim has been a major concern, and undergraduate students were able to reproduce and extend the experiments presented here in different scenarios. More importantly, the social simulation defined here proved to be a powerful tool to gain insights into the emergency plans; e.g., most students detected that the central stairs were the closest exit to nearly all rooms. Therefore, providing all users with the shortest path to an exit at the same time causes dangerous collapses, whereas the other two alternatives are underused.

## 7. Conclusion and Future Works

After explaining the interest in enhancing emergency plans by ambient intelligence and the necessity of simulation techniques to evaluate these plans, this paper has linked to over 150 different simulators capable of conducting some aspects of this type of assessment, but, as explained, not all. Furthermore, thousands of projects that, to some extent, address this problem have been found. Inasmuch as most of the research literature focuses on improving emergency services, the simulators and simulations employed to evaluate these contributions often remain obscure; *i.e.*, the code or the implementation are not available or the experiments are not described enough, for example. This fact forces new researchers to build an evaluation framework from scratch, which can be even more complex than the service to be deployed and assessed.

To improve this situation, this research proposal paper has presented a holistic framework that attempts to address all emergency evaluation aspects and that facilitates the reuse, extension and interconnection of its major components. The framework has noted the necessity of: (1) including modules that formalize the concepts involved in the remaining modules by using semantic web technologies; (2) offering abstract models prior to the implementation without specific programming language constraints by employing the agent-based simulation paradigm; and, when implementing these models; (3) giving an open and highly modularized source code to enhance its reuse and extension in the context of a service-oriented architecture.

The paper has also described the methodological framework required for this novel combination of technologies, which emphasizes the integration of previous works and the diffusion of each little advance in the construction of this holistic framework. Finally, this work contributes an open and free software tool called EscapeSim [78], which is based on the also-free software, UbikSim [75]. The use of this software has been illustrated in the context of evaluating a number of emergency plans in a hotel (a presentation video [79] and the experimental results are available on-line [81]). EscapeSim intends to be a first grain of sand in the construction of the complex framework described here and an example of code dissemination.

Future work should focus on revising evacuation algorithms in the specialized literature, adapting the algorithms to make them contemplate the main elements considered in the holistic framework presented (particularly, an imperfect vision of the world given by sensors and the necessity of actuators to establish contact with users) and evaluating the performance of the existing algorithms and new adaptations proposed by using simulation techniques.
